# The Use and Abuse of Potassa Fusa and Potassa Cum Calce in the Treatment of Uterine Diseases

**Published:** 1854-10

**Authors:** Henry Bennet


From the London Lancet.

*The Use and Abuse of Potassa Fusa and Potasa Cum Calce in the treatment of Uterine Diseaset.* By Henry Bennet, M. D.

More than nine years ago, Dr. Bennet first introduced potassa fusa and potassa cum calce as valuable agents in the treatment of uterine inflammation. They had since been used by many practitioners; but, he believed, not always with the caution which was imperatively required; and he was therefore anxious to lay down even more carefully than before, the rules which ought to be attended to in resorting to so powerful an agent. He had obtained cylinders of potassa cum calce, in the proportion of two parts of potash to one of lime, which did not deliquesce, and were nearly as manageable as nitrate of silver, and therefore free from many of the objections urged against potassa fusa. The conditions of local uterine disease in which he considered that caustic potash, or the actual cautery was applicable, were the following: Chronic inflammation or inflammatory ulceration of the mucous membrane covering the cervix, or lining its cavity, intractable to other treatment; chronic inflammatory hypertrophy of the cervix, also intractable to other means; and lastly, chronic inflammation of the body of the uterus, in which form of disease the potash is merely applied to the cervix to produce a derivative issue, as we would apply an issue to the back in disease of the spine.

In the first class of cases, the caustic potash is used to modify morbid vitality of the diseased tissues, and to substitute a healthy reparative action. In cases of hypertrophy, the elimination of a moderate sized eschar on the enlarged cervix is attended with acute congestion or inflammation of the subjacent hypertrophied tissues; and under its influence the latter soften and melt, as it were, or are absorbed. The object was not to destroy the enlarged cervix, but to procure its absorption. When applied to the cervical canal, the caustic potash reached the mucous follicles concealed between the rugæ of the *arbor vitæ*, which are occasionally the seat of chronic inflammation, and resist every other means of treatment. Although caustic potash was one of the most valuable contributions ever made to uterine pathology, Dr. Bennet considered it an *ultima ratio*, a last resource, only to be employed when all other means of treatment, general and local, had failed If used cautiously, there was no danger whatever incurred by the patient; but if incautiously or imprudently employed, serious results might follow. The inflammation produced in the cervix might pass to the uterus. The vagina had also been compromised by the extension of the caustic; and in several cases, the potash having been employed too freely to the cervical canal, the os or canal had been nearly obliterated by subsequent contraction and adhesion of its parietes. To prevent these accidents, he advised practitioners never to attempt to destroy the hypertrophied cervix, as had been proposed, but to be satisfied by producing the eliminatory inflammation already described, to use the potassa cum calce cylinders which do not deliquesce; and when the potassa is applied to the cervical canal, to apply it very gently, and to pass a bougie through the canal once or twice a week for six weeks.—Dr. Bennet never resorted to this treatment in passive hypertrophy, either of the cervix or uterus, which he thought might be left to nature, time and general treatment. It had been often stated that the use of caustic potash to the cervix left indurated cicatrices, which might impede subsequent labors. This was by no means the case. So far from producing induration, this mode of treatment removed it, and facilitated parturition. Indeed, he was becoming more and more convinced of the truth of a statement he had made many years ago, that rigidity of the os in labor was nearly always the result of previous inflammatory diseases.

